# Decreased Insulin Secretion but Unchanged Glucose Homeostasis in Cadmium-Exposed Male C57BL/6 Mice

**DOI:** 10.1155/2019/8121834

**Published:** 2019-06-20

**Authors:** Xiaoyin Li, Mengyang Li, Jiming Xu, Xiang zhang, Wei Xiao, Zengli Zhang

**Affiliations:** School of Public Health, Soochow University, 199 Ren'ai Road, Suzhou 215123, China

## Abstract

Cadmium (Cd) is a well-known toxic metal element that is largely distributed in the environment. Cd causes toxicity to most organs. Accumulating evidence suggests that Cd exposure is associated with islet dysfunction and development of diabetes, but the association remains controversial. The aim of this study is to evaluate the possible effects of chronic Cd exposure on glucose metabolism in male C57BL/6 mice. Mice were intraperitoneally injected with CdCl_2_ solution (1 mg.kg^−1^) twice a week for 24 weeks. Fasting blood glucose (FBG) levels and body weights were measured weekly. After 24 weeks, the intraperitoneal glucose tolerance test (IPGTT), intraperitoneal insulin tolerance test (IPITT), and fasting serum insulin (FSI) level test were performed. The insulin resistance index (HOMA-IR) and pancreatic *β* cell function index (HOMA-*β*) were calculated and analyzed. The expression of insulin receptor (IR) in mouse liver was detected by real-time PCR. Pancreatic tissue was collected for histological examination. The results demonstrated that FBG, IPGTT, HOMA-IR, and HOMA-*β* were identical between Cd exposure and control mice. In contract, mean fasting serum insulin level, area under the curve (AUC) of IPITT, and IR expression in livers of Cd-exposed mice decreased significantly compared with control mice. Cd administration induced islet atrophy and decreased islet area. The results suggested that Cd exposure decreased insulin secretion and maintained glucose homeostasis in male C57BL/6 mice and that pancreatic functions should be monitored in populations chronically exposed to Cd.

## 1. Introduction

Diabetes mellitus (DM) is a major contributor to global mortality and disability. The incidence of DM is rapidly growing in both developing and the developed countries [1, 2]. Both genetic and environmental factors contribute to DM development by inducing insulin resistance and *β*-cell dysfunction [3, 4]. However, changes in the genetic factors cannot account for the rapid increase in prevalence of DM in recent decades. Therefore, environmental factors are likely the key to understanding the epidemic [5, 6].

Cadmium (Cd) is a metal element found in the earth's crust and is largely present in the environment, especially in industrial and urbanized regions. Cd is a nonessential trace element for human metabolism. However, a low-level Cd exists in the general population [7]. Individuals can be easily exposed to Cd through smoking, contaminated food or water, and occupational exposure [8]. It was reported that Cd exposure resulted in damage to the lung, liver, testes, kidney, and bone tissues [9]. Recently, Cd was recognized as an endocrine disruptor in humans and rodents [10, 11]. A growing concern is the impact of Cd exposure on the development of DM. Epidemiological and experimental studies investigated the possible association between Cd exposure and incidence of DM [7–11]. These studies have shown Cd-induced changes in islet function, insulin secretion, and insulin activity that affected blood insulin levels and glucose metabolism [10–12]. It was reported that fasting serum insulin levels were significantly decreased in Cd-exposed smelter workers compared with non-Cd-exposed workers [13]. However, the exact mechanism of Cd-induced disruption of glucose homeostasis is unknown [14, 15]. To further study the topic, the effects of long-time low-dose Cd exposure on glucose homeostasis were investigated in male C57BL/6 mice.

## 2. Materials and Methods

### 2.1. Animal Studies

Eight-week-old male C57BL/6 mice were provided by the Animal Center of the Soochow University, as recommended by the responsible local animal ethics review board. All mice were housed in groups of five at 23 ± 2°C on a 12-h light and dark cycle. They were provided with a standard chow diet and water ad libitum. Food was only withdrawn as required for fasting.

The mice were randomly divided into two groups: control (Con mice) and chronic Cd-exposed mice (Cd mice). Each group contained 10 mice. Cd group mice were injected intraperitoneally with 1 mg/kg, twice weekly CdCl_2_ (Gexin Chemical Plants, Wuxi, China) for 24 weeks, and control mice were injected with normal saline (0.9% NaCl).

At the end of the experimental period, blood was collected into plain tubes without anticoagulant by cardiac puncture under general anesthesia (10% chloral hydrate) after overnight fasting. Mice were sacrificed thereafter by cervical dislocation. The blood was allowed to clot at room temperature for 10 minutes and then centrifuged at 3000 ×g at 4°C for 10 min to obtain serum. Serum was stored at -20°C until analyzed for insulin. The pancreas was removed and placed into 4% neural buffered formalin for fixation. Other organs were removed, weighted, and stored frozen at -80°C.

### 2.2. Analytical Procedures

Blood for glucose levels was collected from a tail vein and measured using an ACCU-CHEK Performa kit (Roche Diabetes Care, Mannheim, Germany). Serum insulin concentrations were measured using mouse insulin ELISA kit (F5618-B, SANJIA, China).

### 2.3. Intraperitoneal Glucose Tolerance Test (IPGTT)

All mice were fasted on the first night of 24th week. IPGTT was performed the next morning. Mice were injected with a glucose solution (2 g/kg body weight; D-(+)-Glucose, Sinopharm Group Company, Shanghai, China) and blood glucose levels were measured at 0, 15, 30, 60, and 120 min after the glucose load.

### 2.4. Intraperitoneal Insulin Tolerance Test (IPITT)

On day six of the 24th week, IPITT was performed. Mice were fasted for 6 hours and human regular insulin (0.75 U/kg body weight) (Wanbang, China) was injected intraperitoneally. Blood glucose levels were measured at 0, 15, 30, and 60 min after insulin injection.

### 2.5. Insulin Resistance Index

The HOMA-IR index (homeostatic model assessment of insulin resistance, HOMA-IR) and HOMA *β*-cell index (homeostatic model assessment of *β*-cell index, HOMA-*β*) were calculated from the fasting concentrations of insulin and glucose to estimate the insulin resistance and *β* cell function [16, 17]. The equations used were the following: HOMA-IR= (FSI×FBG) ÷22.5; HOMA-*β*= (20×FSI) ÷ (FPG-3.5). FSI is fasted serum insulin concentration (mU/L) and FBG is fasting blood glucose (mmol/L).

### 2.6. Histological Examination

Pancreas tissue samples were fixed in 4% neural buffered formalin, dehydrated, and embedded in paraffin. Embedded tissues were sectioned at 5-*μ*m thickness and stained with hematoxylin and eosin (H&E). Histological features were observed using light microscopy (CKX41, OLYMPUS, Tokyo, Japan). At least 25 different areas each pancreas slide were observed, and all islets were counted and measured using Image Pro Plus 6.0.

### 2.7. Real-Time/Quantitative Polymerase Chain Reaction (PCR)

Total RNA was extracted from livers with Trizol reagent (Takala). The RNA concentration was determined using a NanoDrop 2000c, Thermo. The RNA was reverse transcribed to cDNA using Hifair II 1st Stand cDNA Synthesis SuperMix (Yeasen). Real-time PCR amplification of the insulin receptor (IR) was performed using the SYBR Green Master Mix (Yeasen). Samples were run as duplicates for 40 cycles using Applied biosystems, 7500. Before the cDNA amplification, it was predenatured at 95°C for 5 min. PCR conditions were 40 cycles of denaturation at 95°C for 50 s, annealing, and primer extension at 60°C for 30 s. Cycle thresholds were measured and the relative expression of IR gene was calculated by comparison of Ct values. Primers were designed from the respective gene sequences from Pubmed. Sequences of the primers were as follows: *β*-actin, 5′-TGCGCCTGCAGAGATTCAAG-3′ (forward) and 5′-AGGTAACGCCAGGAATTGTTGCTA-3′ (reverse); IR, 5′-TTTGTCATGGATGGAGGCTA-3′ (forward) and 5′-CCTCATCTTGGGGTTGAACT-3′ (reverse).

### 2.8. Statistical Analysis

Data are presented as mean ± SEM. Data were entered into Excel 2011 and analyzed for statistical significance using one-way analysis Student's* t* test or Mann-Whitney U test. Statistical analyses were performed using SPSS 16.0 software (SPSS).* P* values<0.05 were considered significant.

## 3. Results

### 3.1. Body Weight and Body Weight Gains

**Figure 1:**
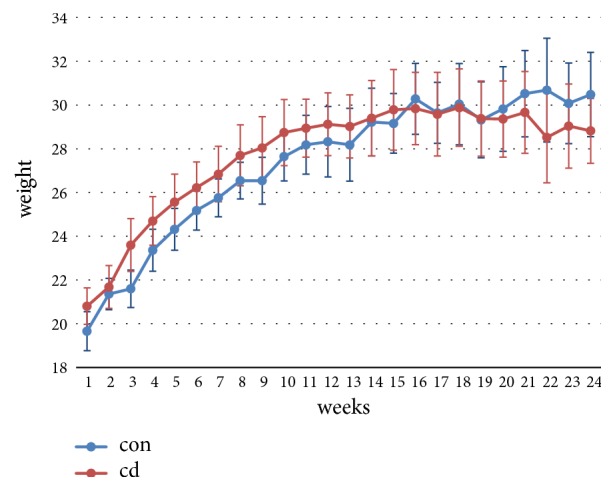
Effects of Cd (cadmium chloride; 1 mg.kg^−1^, intraperitoneal injection, twice weekly) on body weights in male C57BL/6 mice. Cd exposure had no significant effect on body weight in male C57BL/6 mice, n=5/group.

In the present study, exposure to Cd had no significant effect on body weight or body weight gain in mice. The mean body weights of all mice were essentially unchanged after 12th week as shown in Figure 1.

### 3.2. Chronic Cd Exposure Decreased Insulin Secretion in Mice

**Figure 2:**
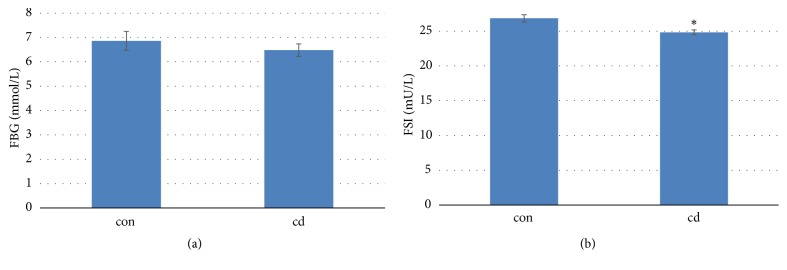
Effects of chronic Cd exposure on FBG and FSI (FBG: (a); FSI: (b)) levels in male C57BL/6 mice. Data are mean ± SEM (n=5/group). *∗*Significantly different from con and Cd groups, at* P<*0.05.

All the mice were fasted overnight and the fasted glucose and insulin at 24th week were measured. Chronic Cd exposure had no effect on fasted blood glucose (Figure 2(a)). However, Cd-exposure significantly decreased fasted insulin levels in the treated mice (Figure 2(b)). The decreased insulin did not cause the changes of glucose levels in male C57BL/6 mice.

### 3.3. Chronic Cd Exposure Did Not Change Insulin Resistance in Mice

**Figure 3:**
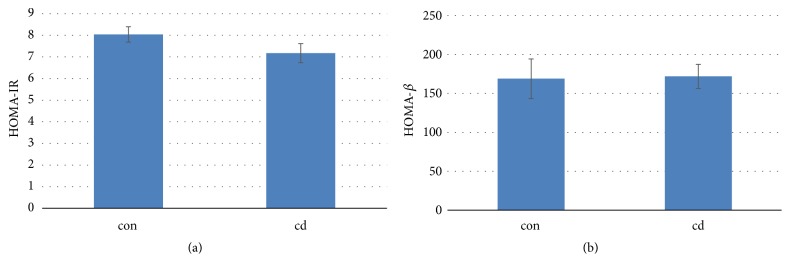
Effects of chronic Cd exposure on the insulin resistance ((a): HOMA-IR; (b): HOMA-*β*) in male C57BL/6 mice. Data are mean ± SEM (n=5/group).

**Figure 4:**
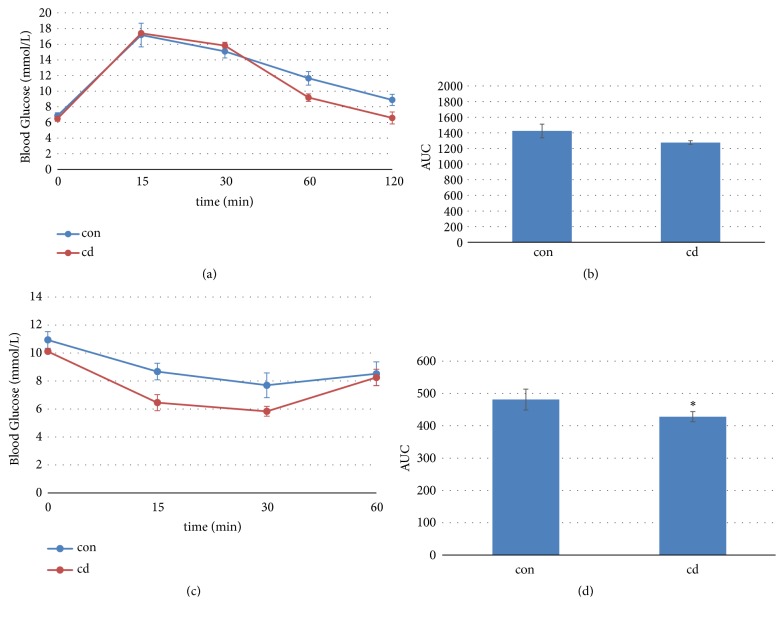
Effects of chronic Cd exposure on the intraperitoneal glucose tolerance test (IPGTT: (a) and (b)) and the intraperitoneal insulin tolerance test (IPITT: (c) and (d)) in male C57BL/6 mice. Data are mean ± SEM (n=5/group). *∗*Significantly different from con and Cd groups, at* P<*0.05.

There were no significant differences in HOMA-IR and HOMA-*β* between the two groups (Figure 3). Chronic Cd exposure did not increase the insulin resistance in mice. The area under the curve (AUC) of IPGTT was identical in the two groups' mice (Figures 4(a) and 4(b)). AUC of IPITT in Cd-exposure mice decreased significantly compared to that in control mice (Figures 4(c) and 4(d)).

### 3.4. Chronic Cd Exposure Induced Islet Atrophy in Pancreas

**Figure 5:**
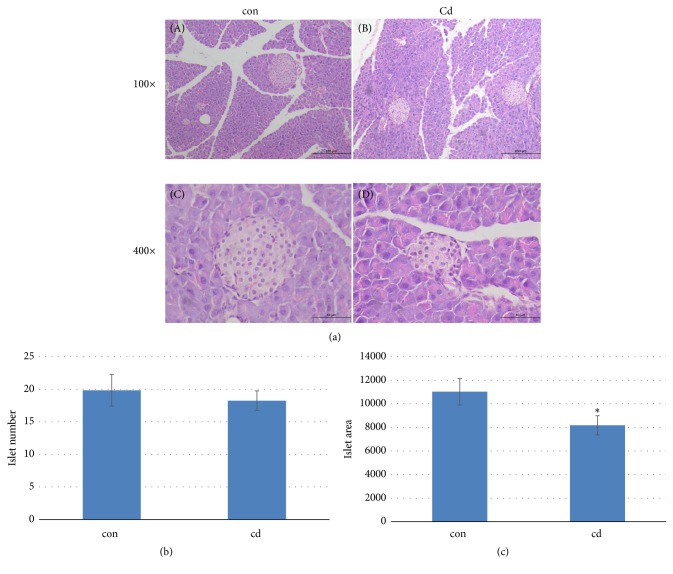
Histopathological evaluation of chronic Cd exposure (cadmium chloride; 1 mg.kg^−1^, intraperitoneal injection, twice weekly) on pancreas tissue in male C57BL/6 mice (n=5/group) (a): (A), (B): 100×; (C), (D): 400×. Tissue samples were fixed in 4% neutral buffered formalin, embedded in paraffin, sectioned, and stained with H&E. (A), (C): pancreas tissue of control mice; (B), (D): pancreas tissue of chronic Cd-exposed mice showing islet atrophy. (b): islet numbers in control and Cd-exposed mice. (c): the areas of pancreatic islets in control and Cd-exposed mice.

Histopathological evaluation demonstrated that control group pancreas sections had normal architecture (Figures 5(a)(A) and 5(a)(C)), whereas pancreas tissue from chronic Cd-exposed mice showed islet atrophy (Figures 5(a)(B) and 5(a)(D)). The number of islets in the pancreas sections was counted and there were no differences between the two groups (Figure 5(b)). However, the mean area of islets measured by Image Pro Plus in the Cd group was significantly decreased compared with control group mice (Figure 5(c)).

### 3.5. Chronic Cd Exposure Decreased the Expression of the Insulin Receptor Gene in Mouse Liver

**Figure 6:**
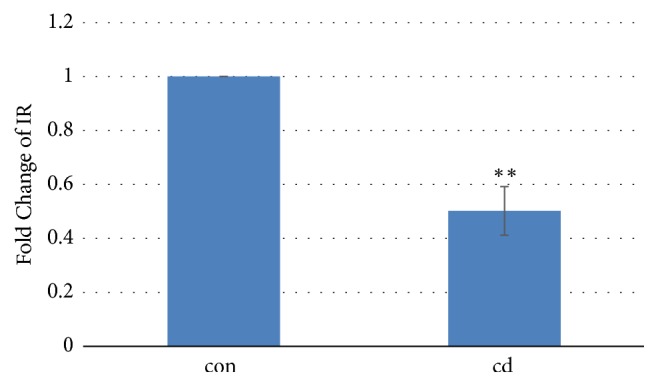
Effects of Cd (cadmium chloride; 1 mg/kg, intraperitoneal injection, twice weekly) on mRNA levels of IR in mouse liver, n=5/group. *∗∗*Significantly different from con and Cd groups, at P<0.01.

Real-Time-PCR showed that IR mRNA levels in the liver were decreased significantly (p<0.01) in Cd-exposed mice. The liver IR gene-expression data showed an approximately 50% decrease in Cd exposed mice compared with the control group (Figure 6).

## 4. Discussion

DM incidence is increasing worldwide. Accumulating evidence has revealed that environmental pollutants may play a critical role in this increasing [18, 19]. The present study evaluated the effects of chronic Cd exposure on glucose homeostasis in mice.

After 24 weeks of Cd exposure, FBG levels in Cd-exposed mice were identical to control mice. Cd exposure did not change glucose homeostasis in treated mice, which was further confirmed by IPGTT analysis. These results are consistent with a population study that found that Cd exposure was not associated with increased risk of type 2 diabetes [20]. There are conflicting data about Cd-induced hyperglycemia. Other studies have suggested a possible link between Cd exposure and altered glucose metabolism resulting in diabetes-like hyperglycemia [19]. Several animal studies reported that Cd exposure induced hyperglycemia [21, 22]. However, the experimental conditions in these studies were different from this study. Firstly, rats were used as animal model in other studies while mice were used in our study. It is possible that mice have better compensatory functions than rats in glucose homeostasis. This could explain why Cd-exposed mice maintained normal glucose homeostasis despite decreased insulin level. Secondly, other studies were acute or subchronic exposure models. The Cd-exposure durations in other studies were much shorter compared with this study [22, 23]. To our knowledge, the present study is the longest duration study Cd exposure. Different species and durations of Cd exposure may have contributed to the different results.

In contrast to the unchanged FBG level, Cd-exposure significantly decreased the fasting serum insulin level in mice, which is consistent with previous studies [21, 22]. Distribution of Cd was not analyzed in our study, which is a limitation of our study. The pancreas is one of the major sites of Cd accumulation [24, 25]. Therefore, the reduction of fasting insulin levels in Cd-exposed mice suggests a possible direct toxic effect of Cd on the pancreas. Histological examination revealed a significant decrease in the relative area of the islets in Cd-exposed mice. The reason for the discrepancy between decreased insulin secretion and unchanged glucose homeostasis is unclear. It is possible that the changes in FSI and islet atrophy in Cd-dosed mice were too small to have a physiological effect on glucose homeostasis. There also could be other reasons that require future investigation.

What is more interesting is that Cd-exposure significantly decreased AUC of IPITT in present study. The reason for this may be that baseline insulin levels in Cd-exposed mice were less than control mice. Therefore, exogenous insulin may have greater effect in Cd-exposed mice, meaning that Cd exposure did not impair tissue sensitivity to insulin. The results of both HOMA-IR and HOMA-*β* also confirm the unchanged insulin sensitivity. However, epidemiological studies found significant correlations between Cd exposure and decreased insulin sensitivity [26, 27]. It has been reported that insulin insensitivity is characterized by defective insulin receptor (IR) signaling and IR downregulation is a well-established contributor to insulin insensitivity [28, 29]. However, significant IR downregulation in Cd-exposed mouse liver did not result in insulin insensitivity in the current study. To date, the topic remains ill-defined.

There are two limitations in the present study. Firstly, only one Cd exposure group was studied and the dose-response relationship of Cd could not be investigated. Secondly, the Cd content of organs was not determined. Several studies reported that the pancreas is one of the target organs of Cd and Cd exposure produced a time-dependent Cd accumulation in the pancreas [16, 30, 31]. Substantial Cd concentration in the pancreas in present long-term Cd exposure study is speculated. In this long-term Cd-exposure study, Cd-exposed mice did not show any change in body weight and obvious toxicity. It indicates that the Cd levels administered to the mice in current study were reference to environmental exposure level.

In conclusion, current study demonstrated that chronic Cd exposure decreased insulin secretion but maintained glucose homeostasis in male C57BL/6 mice, which supplemented current knowledge on the relationship between low Cd exposure and pancreatic toxicity. Further study about the effects of Cd exposure on insulin sensitivity and glucose homeostasis is warranted.

## Data Availability

The data used to support the findings of this study are included within the article.
